# The genus *Pterostichus* in China III: a brief review of subgenus *Chinapterus* Berlov (Coleoptera, Carabidae) with descriptions of two new species

**DOI:** 10.3897/zookeys.953.52282

**Published:** 2020-07-27

**Authors:** Sodnomtsog Dorjderem, Hongliang Shi, Hongbin Liang

**Affiliations:** 1 Key Laboratory of Zoological Systematics and Evolution, Institute of Zoology, Chinese Academy of Sciences, Beijing 100101, China Chinese Academy of Sciences Beijing China; 2 College of Life Science, University of Chinese Academy of Sciences, Beijing 100049, China University of Chinese Academy of Sciences Beijing China; 3 College of Forestry, Beijing Forestry University, Beijing 100083, China Beijing Forestry University Beijing China

**Keywords:** China, *
Pterostichus
*, *
Chinapterus
*, new synonym, key

## Abstract

The subgenus Chinapterus Berlov, 1998 of the genus *Pterostichus* is briefly reviewed and a new synonym is proposed: *Pterostichus singularis* Tschitschérine, 1889 = *Pterostichus balthasari* Jedlička, 1937 **syn. nov.** Two new species are described: Pterostichus (Chinapterus) lianhuaensis**sp. nov.** and Pterostichus (Chinapterus) liupanensis**sp. nov.***Pterostichus przewalskyi* Tschitschérine, 1888 is moved from subgenus Sinoreophilus Sciaky, 1996 to *Chinapterus*, and lectotypes are designated for *P. balthasari* and *P. przewalskyi*. A key to four species of the subgenus Chinapterus is provided.

## Introduction

The subgenus Chinapterus was erected by Berlov (1998) based on type species of *Pterostichus balthasari* Jedlička, 1937 from Gansu, China. This small subgenus is endemic to midwestern China. Recently, when sorting carabid specimens at the Institute of Zoology, Chinese Academy of Sciences, we found eight specimens from Lianhua Shan in Gansu, and three specimens from Liupan Shan in Ningxia belonging to this subgenus. After comparison with type specimens of *Chinapterus*, we believe they represent two new species. Furthermore, we also found that the type species, *P. balthasari* is a junior synonym of *Pterostichus singularis* Tschitschérine, 1889, and that another species, *Pterostichus przewalskyi* Tschitschérine, should be included in the subgenus Chinapterus. Therefore, we would like to make a brief review of species belonging to the subgenus Chinapterus in this paper.

## Materials and methods

This paper is mainly based on the examination of specimens from China. The majority of specimens examined, including types of new species, are deposited in the collection of the Institute of Zoology, Chinese Academy of Sciences (**IZAS**). The specimens examined or cited from other collections are indicated with abbreviations as follows.

**MNHN** Muséum National d’Histoire Naturelle, Paris, France.

**MSNM**Museo Civico di Storia Naturale, Milano, Italy.

**NMPC**Národní Muzeum Přírodovědecké Muzeum, Prague, Czech Republic.

**ZIN**Zoological Institute, Russian Academy of Sciences, St. Petersburg, Russia.

The body length (BL) was measured from the apical margin of the labrum to the elytral apex; the body width (BW) was measured along the elytral greatest width (EW). The metepisternum length (ML) was measured along the inner margin; width (MW) was measured along the basal margin. The pronotum width (PW) was measured along its greatest width; basal width (PBW) was measured along its basal margin; apical width (PAW) was measured along its apical margin, pronotum length (PL) was measured along its median line. Elytra length (EL) was measured along the suture from the base of the scutellar to the elytra apex. For the description of the endophallus, all lobes were named based on their homological inferences but not actual locations. The abbreviations used in the endophallus are as follows: gonopore (gp), gonopore lobe (gpl), gonopore piece (gpp), dorsal lobe (dl), left basal lobe (lbl), left apical lobe (lal), and right lobe (rl). Other terms used, dissection techniques, endophallus everting procedures, and photography are consistent with what we adopted in our previous work (Shi et al., 2013; Shi & Liang, 2015). Original labels are cited for types and non-types.

## Taxonomy

### Key to subgenera of the genus *Pterostichus* from China (part)

**Table:** 

1	Mesofemur with four or more setae along posterior margin; spermatheca tubular, seminal canal and receptaculum not differentiated, spermathecal canal inserted between midpoint to the apical third of spermatheca	**2**
–	Mesofemur with two (occasionally three) setae along posterior margin; spermatheca usually with seminal canal and receptaculum differentiated, seminal canal more or less slenderer than receptaculum, OR spermatheca short, seminal canal and receptaculum not differentiated, spermathecal canal inserted near basal fourth of spermatheca	***Pterostichus* other subgenera**
2	Metepisternum long, length much greater than the width of anterior margin; hind wing well developed	**3**
–	Metepisternum short, length subequal to the width of anterior margin; hind wing reduced	**4**
3	Pronotum strongly cordate; lateral region between lateral bead and basal fovea flat; apical setigerous pore on the third elytra interval near apical twelfth	**sg. Adelosia**
–	Pronotum subquadrate or a little narrowed to the base; lateral region between lateral bead and basal fovea more or less ridged; apical setigerous pore on the third elytra interval near apical sixth	** sg. Platysma**
4	Elytral striae complete	**5**
–	Elytral striae tangled, interrupted, sinuate or replaced by rows of irregular coarse punctures	**6**
5	Elytral microsculpture isodiametric, similar in both sexes; right paramere falciform, apex somewhat elongate	**sg. Chinapterus**
–	Elytral microsculpture granular in females, isodiametric in males; right paramere rounded triangular, apex not elongate	**sg. Sinoreophilus**
6	Pronotum with two setae near posterior angle; legs bicolor with reddish brown femora	**sg. Plectes**
–	Pronotum with one seta near posterior angle; legs unicolor, black or dark brown	**sg. Metallophilus**

#### Subgenus Chinapterus

Taxon classificationAnimaliaColeopteraCarabidae

Berlov, 1998

9041DD77-0721-5C9C-96D4-C03F7903FA2E

Chinapterus Berlov, 1998: 14. Type species: Pterostichus balthasari Jedlička, 1937 [= Pterostichus singularis Tschitschérine, 1889], by original designation.

##### Diagnosis.

Body slightly convex. Elytral striae regular, third interval with two or three setigerous pores. Metepisternum slightly wider than length. Mesofemur with four or more setae on ventral surface. Elytral plica absent or indistinct. Right paramere of male genitalia falciform, with somewhat elongated apex. Spermatheca with seminal canal and receptaculum not differentiated.

##### Subgeneric characters.

Medium size, body length 9.0–15.0 mm. Black, elytra slightly shiny, without metallic luster. Submentum with two long setae on each side. Pronotum cordate or quadrate; basal foveae deep, inner and outer grooves indistinctly separated or outer groove absent; one baso-lateral seta inserted on basal angle. Elytra striae straight and continuous, neither interrupted nor sinuate; interval microsculpture isodiametric, similar in male and female, third interval usually with two or three setigerous pores, fifth interval without pore; ninth interval with umbilical series slightly sparser in the middle than basal and apical areas; elytral plica indistinct; scutellar stria present; basal pore present or not. Metepisternum slightly wider than length. Terminal ventrite of males slightly depressed or without modification. Mesofemur with four or more setae on ventral surface, with a spine near apex; metacoxae with two setae; metatrochanters without seta. Fifth tarsomere with or without setae on ventral side. Apical orifice of aedeagus obviously twisted to left side; apical lamella narrow, short; right paramere falciform, apex more or less elongate and bent; endophallus bent to ventral-left or venter, gonopore opened to the base, with a large cap-like sclerotized gonopore piece (Figs 7–9). Gonocoxite II of ovipositor stout or slightly slender and bent, apex rounded, inner and outer margin each with one ensiform spine, apex with two very short nematiform setae in groove (Figs 60–65). Spermatheca tube-like, surface glabrous, receptaculum not differentiated from seminal canal, base of seminal canal sclerotized; spermathecal gland very fine, atrium and gland duct not differentiated, connected to the middle of spermatheca (Figs 58, 59).

##### Distribution.

This subgenus is endemic to China. A total of four species are distributed in Qinghai, Sichuan, Gansu, and Ningxia.

##### Comparison.

In its original description (Berlov, 1998), the subgenus Chinapterus was erected based on type species *Pterostichus balthasari* Jedlička, which was formerly a member of the subgenus Euryperis Motschulsky (Jedlička, 1962) and was subsequently synonymized with *Petrophilus* Chaudoir (Kryzhanovskij et al., 1995). *Chinapterus* is similar to *Euryperis* in having metepisternum slightly wider than length, metatrochanters without seta, and pronotum posterior angles widely rounded (only for *P. singularis*). But, *Chinapterus* is quite different from *Euryperis* in the following aspects: (1) mesofemur with four or more setae on the ventral side (with two setae in *Euryperis*); (2) elytral plica absent or indistinct (distinct in *Euryperis*); (3) spermatheca with seminal canal and receptaculum not differentiated (well differentiated in *Euryperis*).

Among the Chinese subgenera of *Pterostichus*, *Chinapterus* is most similar to the subgenera *Sinoreophilus* and *Metallophilus* in external appearance, and having the mesofemur with four or more setae near the hind margin, the metepisternum width subequal to its length, but differs in: (1) right paramere falciform, with the apex more or less elongate and bent; (2) elytral microsculpture isodiametric, similar in both sexes (in the other two subgenera, right paramere rounded triangular, apex not elongate, nor slightly bent; elytral microsculpture granular in females, isodiametric in males). Comparisons with other similar subgenera present in the key to subgenera.

##### Notes on systematics.

Among all subgenera of *Pterostichus* from China, *Chinapterus* is doubtless closely related to other five subgenera (*Platysma*, *Adelosia*, *Metallophilus*, *Sinoreophilus*, *Plectes*) and shares the following important characters: (1) mesofemur with four or more setae along posterior margin; (2) elytral plica absent or indistinct; (3) metatrochanters without seta; (4) spermatheca tube-like, seminal canal and receptaculum not differentiated. These six subgenera form a monophyletic group (the *Platysma* group as defined here) supported by synapomorphic characters 1, 2, and 3 (character polarities discussed in this section follow Bousquet, 1999: 32–36). Except for the Chinese fauna, *Myosodus*, a subgenus centered in the Caucasus, also belongs to this group.

Among the above four characters, characters 1 is exclusive for the *Platysma* group in *Pterostichus* and character 4 is plesiomorphic. The undifferentiated spermatheca is unusual in *Pterostichus* and may suggest a relatively basal position of the *Platysma* group in the genus. So far as we know, all species of the *Platysma* group have a distinctive form of the female reproductive tract: spermatheca tube-like, seminal canal and receptaculum not differentiated, spermathecal canal inserted between the midpoint to the apical third of the spermatheca. In contrast, for most subgenera of *Pterostichus*, the spermatheca is usually very long with the seminal canal and receptaculum differentiated. If not so distinctly differentiated, the seminal canal is at least a little slenderer than the receptaculum, as in the subgenus Orientostichus. Except the Platysma group, only the subgenus Argutor and its relatives (five subgenera from China) have the undifferentiated spermatheca, but their spermathecae are always very short with the spermathecal canal inserted near the basal fourth of spermatheca.

The relationships among subgenera of the *Platysma* group are quite unclear, and even the monophyly of several subgenera is questioned. The subgenera *Platysma* and *Sinoreophilus* can be differentiated by plesiomorphic characters only, while other subgenera are merely defined by one or two apomorphic characters. Except for *Adelosia* (monotypic) and *Plectes* (includes two very closely related species), the monophyly of the other four subgenera are difficult to demonstrate.

We here redefine the subgenus Chinapterus and assign *P. przewalskyi* together with two new species very closely related to it into the subgenus for the following similarities: metepisternum length subequal to its basal width and right paramere falciform, apex rather elongated and bent. These two apomorphic characters may support the subgenus Chinapterus and can clearly differentiate it from all other related Chinese subgenera. However, the monophyly of *Chinapterus* is still questioned, because the elongate right paramere is also present in other subgenera of the *Platysma* group, including part of *Platysma* and all members of *Myosodus*. Moreover, *P. singularis* and *P. przewalskyi* do not look similar in their general appearances, and simply from the shape of right paramere, the former species is more similar to some species of *Platysma* while the latter is more like *Myosodus*. Nevertheless, the present definition of the subgenus Chinapterus is good for convenient taxa recognition at present. Under this, all Chinese *Pterostichus* species with multisetose mesofemora can be assigned to each subgenus. We expected an in-depth phylogenetic study will propose a better and more objective assignment of subgenera in the future.

When the present study on *Chinapterus* was conducted, we examined all *Pterostichus* species belonging to the *Platysma* group from China. We found that, *P. lanista* (Tschitschérine), *P. militaris* (Tschitschérine), and *P. peilingi* Jedlička should be moved into the subgenus Sinoreophilus for the following characters: mesofemur with four or more setae along posterior margin; elytra striae regular; metepisternum short, length subequal to the width of anterior margin; right paramere short and straight.

### Key to species of the subgenus Chinapterus Berlov

**Table:** 

1	Smaller size (9.0–11.0 mm); pronotum nearly quadrate, lateral margins slightly rounded before middle; elytra basal pore usually absent; males without modification on terminal ventrite; right paramere slightly elongate; Qilian mountain range	***P. singularis* Tschitschérine**
–	Larger size (12.0–14.0 mm); pronotum more or less cordate, lateral margins strongly rounded before middle; elytra basal pore always present; male terminal ventrite shallowly depressed and rugose; right paramere strongly elongate	**2**
2	Pronotal basal fovea depressed between inner and outer grooves, forming deep and wide basal fovea; femora reddish brown; elytra third interval usually with two setigerous pores, occasionally with three pores but all adjacent to second stria	***P. przewalskyi* Tschitschérine**
–	Pronotal basal fovea convex between inner and outer grooves, outer groove rudimentary, forming narrow basal fovea; femora black; elytra third interval with three or more setigerous pores, the basal one adjacent to third stria	**3**
3	Pronotum lateral margins strongly sinuate before basal angles; fifth tarsomere with one or two pairs of setae ventrally; apex of right paramere slightly angulate at dorsal-apical end. Gansu, Lianhua Shan	***P. lianhuaensis* sp. nov.**
–	Pronotum lateral margins near straight before basal angles; fifth tarsomere glabrous ventrally; apex of right paramere completely rounded. Ningxia, Liupan Shan	***P. liupanensis* sp. nov.**

#### Pterostichus (Chinapterus) singularis

Taxon classificationAnimaliaColeopteraCarabidae

Tschitschérine, 1889

E974E35E-DEB4-5749-93A3-C5A7B3F80C07

Figs 1–6 , 7–23 , 58 , 64 , 65

Pterostichus singularis Tschitschérine, 1889: 188 (holotype in ZIN; type locality: Amdo); Tschitschérine, 1898: 179 (Feroperis); Jedlička, 1962: 304 (sg. uncertain).
Pterostichus balthasari Jedlička, 1937: 47 (lectotype in NMPC; type locality: Gansu: Liangchow; sg. Euryperis); Jedlička, 1962: 256 (sg. Euryperis); Berlov, 1998: 14 (sg. Chinapterus). syn. nov.

##### Type locality.

*Pterostichus singularis* Tschitschérine, Amdo, pres du fleuve Tay-tong-che (= Qinghai, Datong River). *Pterostichus balthasari* Jedlička, Gansu: Liangchow (= Gansu, Wuwei City).

##### Type examined.

**Figures 1–6.**
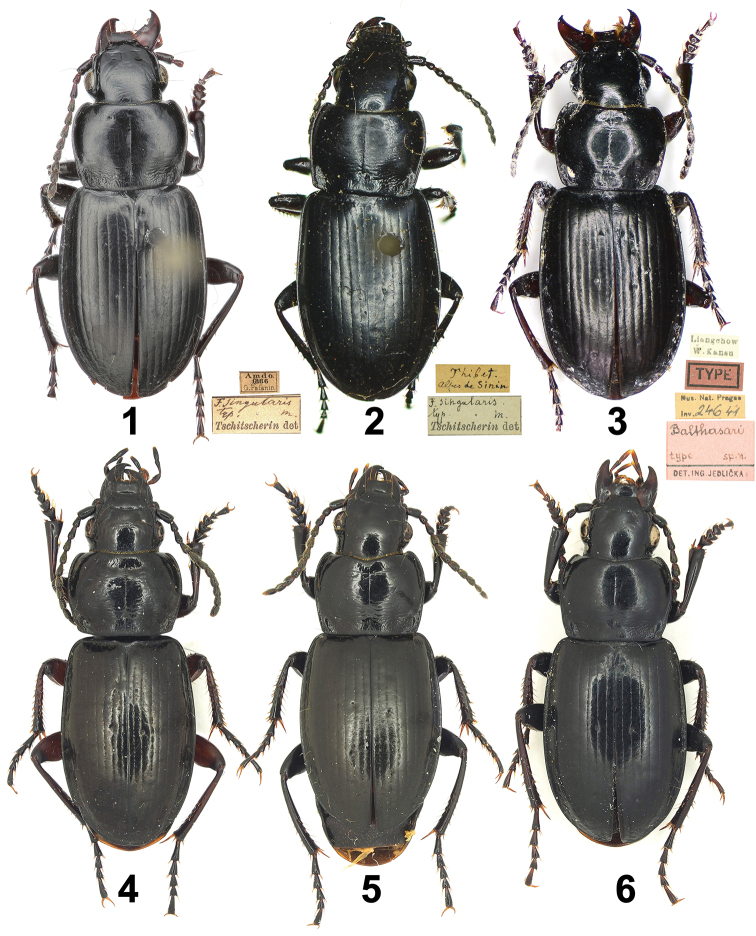
Habitus of Pterostichus (Chinapterus) singularis Tschitschérine. **1** Holotype of *P. singularis* Tschitschérine, male (ZIN) **2** specimen determined by Tschitschérine, female, LT: alpes de Sinin (MNHN) **3** Lectotype of *P. balthasari* Jedlička, male (NMPC) **4** male, LT: Lajishan (IZAS) **5** male, LT: Lenglongling (IZAS) **6** male, LT: Dadongshu pass (IZAS).

***Holotype*** of *Pterostichus singularis* Tschitschérine, male (ZIN) [Figs 1, 22], Amdo, 1886, G. Patani / F. singularis Typ. m. Tschitscherin det; ***Lectotype*** of *Pterostichus balthasari* Jedlička (designated herein), male (NMPC) [Figs 3, 23], Liangchow, W. Kansu / Type [red label] / Mus. Nat. Pragae, Inv. 24641 [orange label] / baltharsari, type, sp. n., Det. Ing. Jedlička [pink label]. ***Paralectotypes*** of *Pterostichus balthasari* Jedlička, 2 females (NMPC), Liangchow, W. Kansu / Cotype [red label] / Mus. Nat. Pragae, Inv. 24642/24643 [orange label] / baltharsari, sp. n., det. Ing. Jedlička.

##### Non-type materials.

**Figures 7–23.**
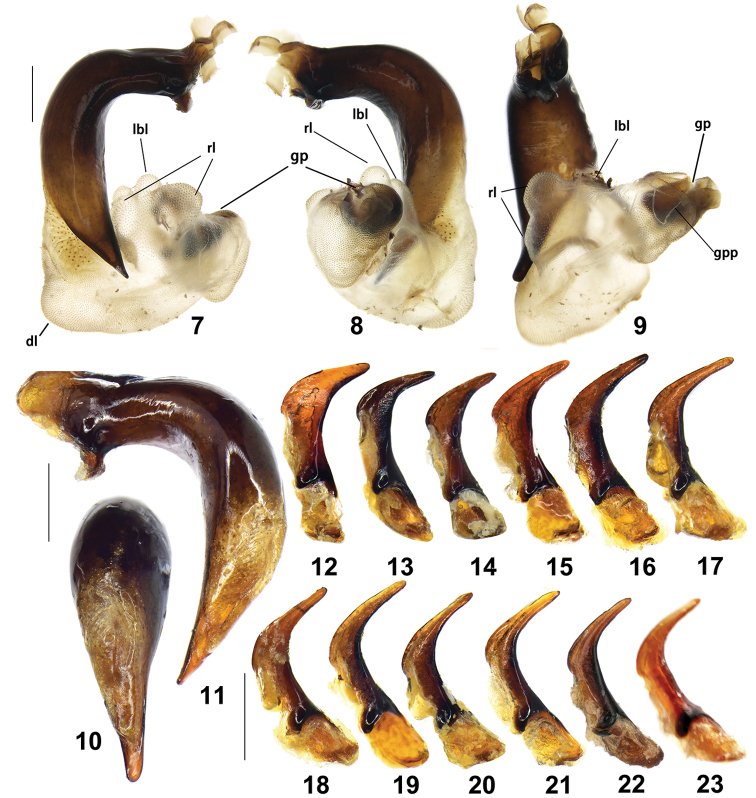
male genitalia of Pterostichus (Chinapterus) singularis Tschitschérine. **7–9** Endophallus, LT: Pass Xining-Guide (IZAS) **7** right lateral view **8** left lateral view **9** ventral view, abbreviations as stated in text **10–11** median lobe of aedeagus, LT: Wushaoling (IZAS) **10** dorsal view **11** left lateral view **12–23** right paramere, inner face **12** LT: Lajishan (IZAS) **13** LT: Lajishan (IZAS) **14** LT: Pass Xining-Guide (IZAS) **15** LT: Guoshizeshan (IZAS) **16** LT: Lajishan (IZAS) **17** LT: E Qilianshan (IZAS) **18** LT: Dadongshu pass (IZAS) **19** LT: Dadongshu pass (IZAS) **20** LT: Lenglongling (IZAS) **21** LT: Wushaoling (IZAS) **22** Holotype of *P. singularis* (ZIN) **23** Lectotype of *P. balthasari* (NMPC). Scale bars: 0.5 mm.

1 female (MNHN) [Fig. 2], Thibet., alpes de Sinin [handwritten] / F. singularis, typ. m., Tschitscherin det [handwritten] / Museum Paris, coll. R. Oberthur. 1 male (MSNM), N slope of Xining mountains near Liandzha-sian, before 19–VI–1890, Gr.-Grzhimajlo leg. [in Russian] / F. singularis, typ. m. Tschitscherin det. / *Paralectotypus*, *Pterostichus*, *Singularis Tschitsch. Vejeschagina 1983* [red label]. 1 female (NMPC), N slope Xining, Liandzha-sian vill., before 19–VI–1890, Grum leg. [in Russian] / *F. singularis*, *typ.*, *m.*, Tschitscherin det / Type [red label] /*singularis*, *typ. Tsch.*, Det. Tschitscherin [pink label]. 5 males and 2 females (IZAS) [Figs 10, 11, 21], Gansu, Tianzhu county, G312 Wushaoling pass; alpine shrub, 37.2057N, 102.8799E, 3307 m, under rock; 2017.VII.12, Shi HL, Lu ZB & Zhu PZ lgt. 1 male and 1 female (IZAS) [Figs 5, 20], Gansu Prov. Lenglong Ling, 80 km NNW Honggu, 3392–3900 m, 37°03'50.3"N, 102°39'57.2"E, alpine pasture with Rhododendron, under stones, 2011.VI.30–31, D.W. Wrase.1 male and 1 female (IZAS) [Figs 4, 13], China, Qinghai Prov. Laji Shan pass, 34 km SSE Huangyuan, 36°23'01"–50"N, 101°19'31"–20'06"E, 3850–3880 m, wet plateau with depression, under stones/ clods, 8.VII.2011, D.W.Wrase. 1 female (IZAS), 2006.7.1–10, Qinghai Prov., Laji Shan. 2 males and 2 females (IZAS) [Figs 7–9, 14, 58, 64, 65], Qinghai, Qilian Shan, 3800m, pass Xi-ning-Guide, 9–11.08.10. M. Murzin. 7 males and 8 females (IZAS) [Figs 12, 16], Qinghai, Guide county, Lajishan pass, alpine meadow, N36.3573 E101.4463, 3829 m, under rock, 2017.VII.20, Shi HL, Lu ZB & Zhu PZ lgt. 2 males and 7 females (IZAS), Qinghai, Guide county, N. Slope of Guoshize Mt.; screes; 4007 m; 36.2914, 101.6005, 2017.VII.21, under stone, SHI HL & LU ZB leg. 1 male and 1 female (IZAS), Qinghai, Guide county, 3 km W of Lajishan pass, alpine meadow, 36.3622N, 101.4117E, 3686 m, pitfall trap; 2017.VII.21; Shi HL, Lu ZB & Zhu PZ lgt. 2 males and 1 female (IZAS) [Fig. 15], Qinghai, Guide county, Guoshizeshan pass, *Salix* shrubs, N36.3082 E101.5973, 3487 m, pitfall trap, 2017.VII.21; Shi HL, Lu ZB & Zhu PZ lgt. 1 male and 2 females (IZAS), Qinghai, Qilian, Datong Shan, 38.020364N, 100.134777E / 3770 m, 2012.V.31, Huang Xinlei leg. 6 males and 1 female (IZAS) [Figs 6, 18, 19], Qinghai, Qilian county, 8 km N of Dadongshu pass, alpine meadow, 38.0531N, 100.2273E, 3541 m, pitfall trap; 2017.VII.18, Shi HL, Lu ZB & Zhu PZ lgt. 1 male (IZAS) [Fig. 17], Qinghai, E. Qilian Shan, 4200 m, 1.8.1992, J.Kalab leg. / Compared with type, SHI HL 2011 / Pterostichus balthasari Jedlička, 1937 / ex coll. Sciaky 2011. 1 male and 1 females (IZAS), Qinghai, Menyuan county, Xianmi, Ningchan pass, alpine meadow, 37.5374N, 101.8704E, 3900 m, under rock, 2017.VII.14, Shi HL, Lu ZB & Zhu PZ lgt. 1 female (IZAS), Qinghai, Menyuan county, Xianmi xiang, Taola, *Picea* forest, 37.2309N, 102.0134E, 2636 m, under rock; 2017.VII.15, Shi HL, Lu ZB & Zhu PZ lgt. 1 female (IZAS), Qinghai, Menyuan county, Xianmi xiang, Bangusi, *Picea* forest, 37.2908N, 101.9438E, 2707 m, pitfall trap; 2017.VII.15, Shi HL, Lu ZB & Zhu PZ lgt. 1 male and 4 females (IZAS), Qinghai, Qilian county, Zhamashi, Zhamashixigou; alpine meadow; 3014 m, 38.1590N, 99.9921E, under rock; 2019.VIII.19; YAN Weifeng lgt. 4 males and 2 females (IZAS), Qinghai, Qilian county, Babao town, Lujiaogou; alpine meadow; 3461 m, 38.0991N, 100.4838E, under rock; 2019.VIII.17; YAN Weifeng lgt. 3 females (IZAS), Qinghai, Menyuan county, Xianmi, Qihankaigou; mixed forest; 2683 m, 37.1568N, 102.0278E, under rock; 2019.VIII.12; YAN Weifeng lgt. 2 males (IZAS), Qinghai, Qilian county, Epu town, Jingyangling pass; alpine meadow; 3716 m 37.8387N, 101.1117E, under rock; 2019.VIII.15; YAN Weifeng lgt. 2 females (IZAS), Qinghai, Qilian county, Babao township, Binggou; alpine meadow; 3826 m 38.1171N, 100.1716E, under rock; 2019.VIII.18; YAN Weifeng lgt. 1 male (NMPC), China: Qinghai province, Gangca Dasi [lamasery]., 37°32.4'–33.0'N, 100°05.3'–06.0'E, 3505–3840 m, 11–12.VII.2005, J. Hajek, D.Kral & J.Ruzicka leg; individually under stones, in excrements and on vegetation; spring; alpine meadows and pastures around the lamasery and in the nearby valley. 1 female (NMPC), China: Qinghai province, Yuning Si [lamasery], 2890 m, 36°45.6'N, 102°10.6'E, 16.VII.2005, J. Hajek, D. Kral & J. Ruzicka leg, individually under stones and logs, in excrements, and on vegetation in coniferous forest, on the pastures, and along the path to a village; pool.

##### Diagnosis.

Femora black. Pronotum quadrate; lateral margins slightly rounded before middle, nearly straight before basal angles; basal angles rounded, or slightly rectangular with an indistinct denticle; basal fovea depressed between outer and inner grooves; elytral basal pore usually absent, third interval usually with two setigerous pores; fifth tarsomere with one or two pairs of fine setae ventrally; right paramere falciform, apex acicular or triangular.

##### Description.

BL 9.2–10.8 mm, BW 3.9–4.4 mm. Robust, black, elytra shiny. ***Head*** large; frons smooth or very sparsely punctate; genae short, less than one-third length of eyes; eyes small, slightly prominent. ***Pronotum*** quadrate; widest before middle, PW/HW = 1.26–1.43, PW/PL = 1.37–1.50; lateral margins slightly rounded from apical angles to the middle, nearly straight before basal angles, mid-lateral seta present at apical third; basal margin slightly wider than apical margin, PBW/PAW = 1.07–1.20; basal angles rounded or slightly rectangular with an indistinct denticle, not protruding outward; basal fovea with inner and outer grooves faintly defined and partly fused, forming deep depression between them, outer groove slightly shorter than inner one; basal foveal area coarsely punctate; middle area between two basal foveae smooth or very sparsely punctate; area between outer groove and lateral margin slightly convex, forming a weak carina; disc convex, smooth, transversely rugose in some specimens. ***Elytra*** oblong, EL/EW = 1.33–1.47; basal ridge slightly oblique; shoulder rounded, basal ridge and lateral margin forming an obtuse angle, humeral tooth absent; apical plica absent; basal setigerous pores usually absent, occasionally present in one elytron or both elytra; scutellar striae short, apex free or connected to first stria; intervals feebly convex; microsculpture similar in both sexes, finely isodiametric; third interval often with two setigerous pores on posterior half, adjacent to second stria, sometimes one or two additional pores present; umbilical series on ninth interval sparse in middle, composed of 14–17 pores; striae moderately deep, distinctly punctate. ***Ventral side.*** Proepisternum sparsely punctate near inner margin, rugose throughout; mesepisternum very sparsely punctate; metepisternum nearly smooth; terminal or penultimate ventrite of males not modified. Tarsomere 5 with one or two pairs of setae ventrally; metatarsomeres without distinct outer furrow. ***Male genitalia.*** Ventral margin of median lobe near straight at middle, slightly bent downwards near apex; apical orifice opened left-dorsally (Figs 10, 11); apical lamella narrowed in dorsal view, length near two folds of basal width, apex rounded. Right paramere falciform, strongly curved, the obtuse angle between basal portion and apical portion 105–110°; apex acicular or narrow triangular, narrow, sharp (Figs 12–21). Endophallus bent to the ventral left side, major portion of endophallus located at left side of median lobe apex; gp opened to basal-left; gpl large and coniform; gpp large and cap-like, on the ventral side of gp; three groups of lobes recognized: dl on dorsal surface of endophallus, gradually swollen; rl on ventral-right surface of endophallus, divided into two conjoint rounded sub-lobs; lbl on the ventral-left surface of endophallus, smaller than rl; lal absent (Figs 7–9). ***Female genitalia.*** Gonocoxite II of ovipositor stout, length ca. 1.5 times maximum width; inner and outer margins arched, each with one ensiform setae near middle, apex rounded, with two very short nematiform setae in a groove (Figs 64, 65). Spermatheca short and tubiform, length ca. four times maximum width (Fig. 58).

##### Distribution.

**Map 1.**
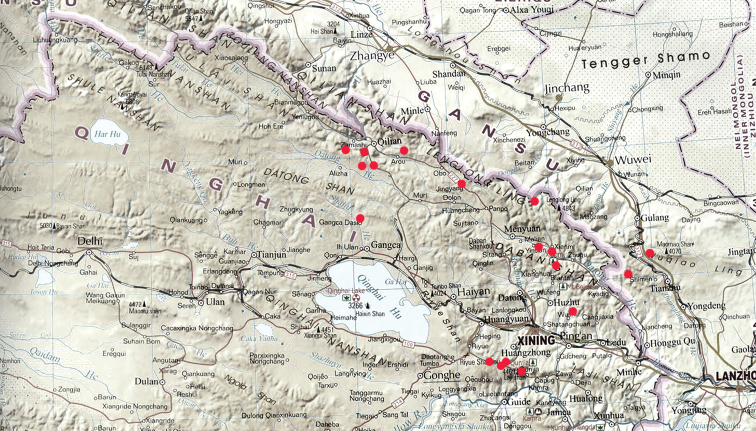
Distributions for *P. singularis*.

Only known from the eastern section of the Qilian mountains on the border of Qinghai and Gansu provinces (Map 1).

##### Habitat.

This species prefers living in open habitat like the alpine meadows of the Qilian mountains between 3000 m and 4000 m, but a few specimens were also found on the edge of *Picea* forest at ca. 2600 m.

##### Remarks.

*Pterostichus singularis* Tschitschérine was described based on a single male from “Amdo, pres du fleuve Tay-tong-che”, referring to what is now the valley of the Datong River in Menyuan County. Thus, the three specimens we examined from different collections are not type specimens but only subsequently determined by Tschitschérine (1898: 181) although labeled as types by the author. The true holotype was deposited in the collection of ZIN.

*Pterostichus balthasari* Jedlička was described based on five specimens from “Liangchow” without an original fixation for holotype. In the collection of NMPC, we examined three specimens in accordance with the original literature. We designate the male bearing a “type” label as the lectotype herein, for the taxonomic purpose of fixing the species name to a single specimen and preventing further confusion. The type locality “Liangchow” refers to what is now Wuwei City in Gansu Province. The exact type locality could be the north slope of Mt. Qilian Shan in Wuwei territory.

Comparing with specimens of *P. singularis* determined by Tschitschérine, the types of *P. balthasari* are nearly identical but the basal angles of the pronotum are more rounded and the type locality is further north. We studied specimens from several localities of the Qilian mountains and found that the northern population (Wushaoling, Lenglongling, Datong Shan) usually has a rounded or a slightly rectangular pronotal basal angle, but the southern population (Laji Shan) generally has a rectangular basal angle of the pronotum;however, a few specimens from Laji Shan also have rounded pronotal basal angles. Moreover, there is no significant difference in the male genitalia between the different localities, both for the median lobe and right paramere. Thus, we synonymize *P. balthasari* with *P. singularis*.

##### Variations.

This species is widely distributed in the eastern section of the Qilian mountain range and is sometimes locally abundant. We studied many specimens from the five branches of the Qilian mountain range: Wushaoling, Lenglongling, Daban Shan, Datong Shan, and Laji Shan. In addition to the above variations of the basal angle, three more variations exist geographically or individually as follows:

(1) elytral dorsal pores on third interval. For ca. two-thirds of the examined specimens, two setigerous pores are present on the third elytral interval. For the other one-third, one or two additional pores are present anterior to the first pore or between the first and second pores.

(2) elytra basal pore. For most specimens there is no basal pore on the elytra, but occasionally, a basal pore is present on one elytron or on both elytra. The proportion of individuals with elytra basal pores is larger in the population from Laji Shan than in the others.

(3) shape of right paramere. The length of the right paramere varies individually as well as geographically. Statistically, the northeastern populations (Wushaoling, Lenglongling, Datong Shan) have a longer right paramere with the apex usually more elongate and acicular as in Figs 17–21. In contrast, the southern (Laji Shan) populations usually have shorter right paramere with the apex less elongate or narrowly triangular as in Figs 12–14. But, this individual variation with a long acicular right paramere is also present in some specimens from the southern localities (Figs 15, 16).

#### Pterostichus (Chinapterus) przewalskyi

Taxon classificationAnimaliaColeopteraCarabidae

Tschitschérine, 1888

FFE05281-4ED0-5340-9306-289B8279B47B

Habitus, Figs 24 , 25 ; male genitalia, Figs 30 , 33 , 42 , 43 , 44 , 47–51 ; female genitalia, Figs 59–61

Pterostichus przewalskyi Tschitschérine 1888: 362 (syntypes in ZIN; type locality: Amdo); Jedlička, 1962: 289 (sg. Oreophilus); Sciaky, 1996: 437 (sg. Sinoreophilus).

##### Type locality.

From the original description, the type localities are “Amdo: près des rivières Tala-tchu et By-tchu, aux sources du Jangtsekiang”. By-tchu refers to the Buqu River in Qumarlêb County of Qinhai Province, and Tala-tchu probably refers to the Derqu River in Chindu County, but the detailed locality was not labeled for any examined type so the precise type locality for the lectotype is unclear.

##### Type series.

**Figures 24–29.**
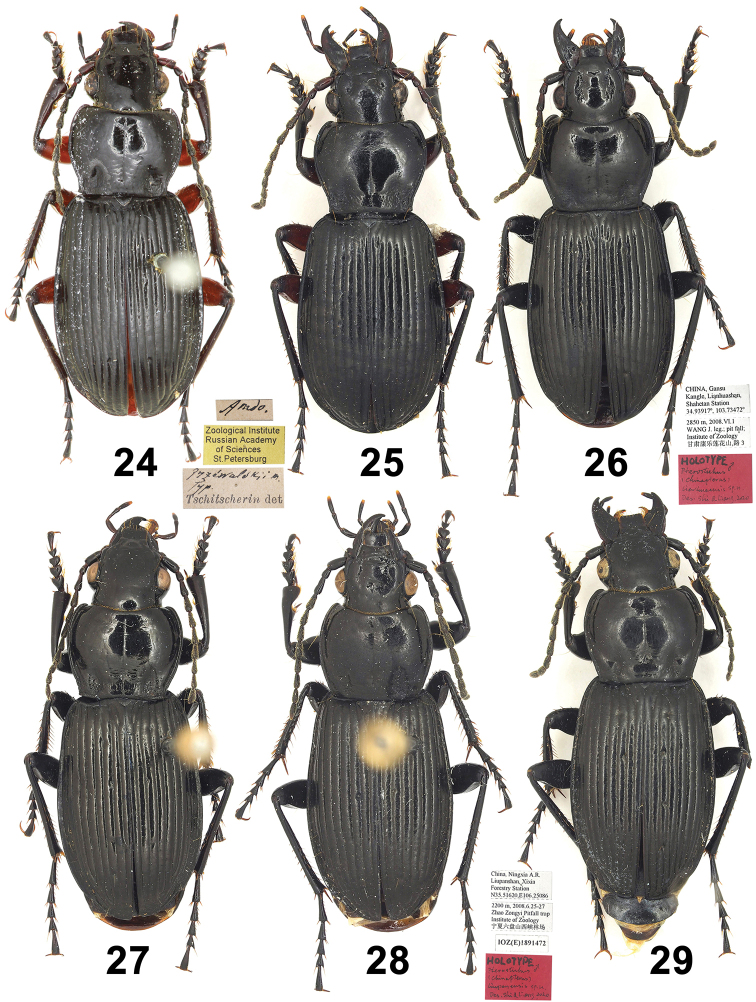
Habitus of Pterostichus (Chinapterus) spp. **24** Lectotype of *P. przewalskyi*, male (ZIN) **25***P. przewalskyi*, male, LT: Hongxing (IZAS) **26** Holotype of *P. lianhuaensis* sp. nov. (IZAS) **27** Paratype of *P. lianhuaensis* sp. nov., male (IZAS) **28** Holotype of *P. liupanensis* sp. nov. (IZAS) **29** Paratype of *P. liupanensis* sp. nov., male (IZAS).

**Figures 30–41.**
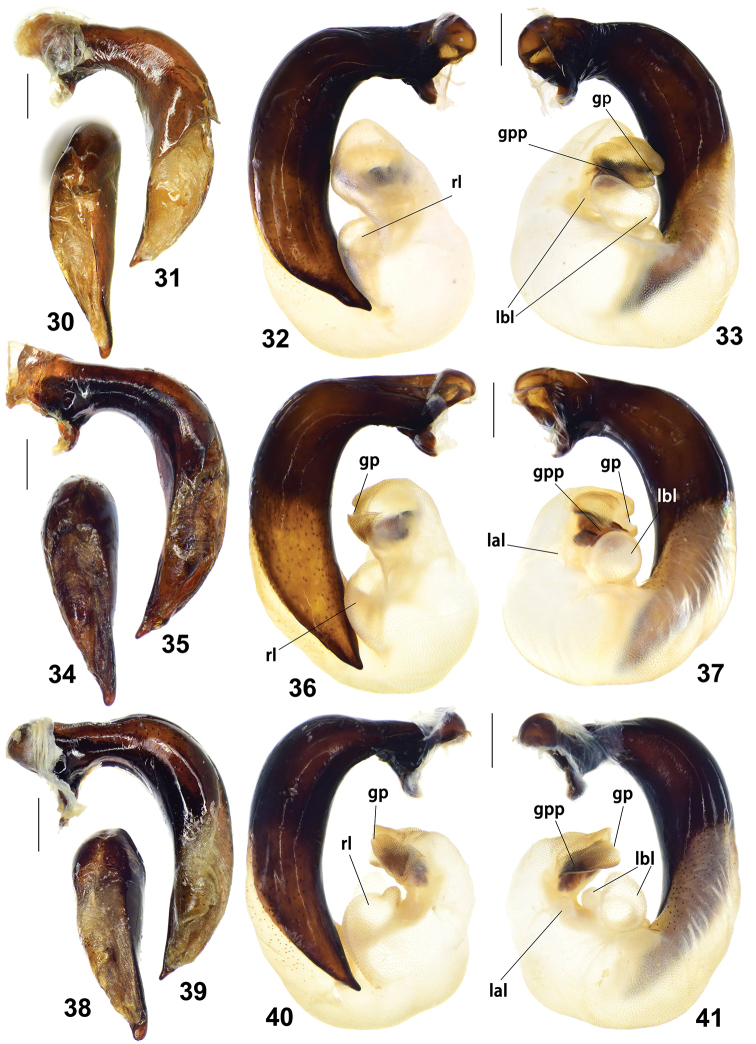
male genitalia of Pterostichus (Chinapterus) spp. **30–33***P. przewalskyi*; 30–31 median lobe of aedeagus, dorsal and left lateral view, Lectotype (ZIN) **32, 33** endophallus, right and left lateral view, LT: Jigzhi (IZAS) **34–37***P. lianhuaensis* sp. nov. **34, 35** median lobe of aedeagus, dorsal and left lateral view, Holotype (IZAS) **36, 37** endophallus, right and left lateral view, Paratype, LT: Lianhuashan (IZAS) **38–41***P. liupanensis* sp. nov. **38, 39** median lobe of aedeagus, dorsal and left lateral view, Holotype (IZAS) **40, 41** endophallus, right and left lateral view, Paratype, LT: Liupanshan, Xixia (IZAS). Scale bars: 0.5 mm, abbreviations as stated in text.

***Lectotype*** of *Pterostichus przewalskyi* Tschitschérine (designated herein), male (ZIN) [Figs 24, 30, 31, 47], Amdo / Przewalskii m. Typ. Tschitscherin det / Zoological Institute Russian Academy of Sciences, St. Petersburg [yellow label]. ***Paralectotypes*** of *Pterostichus przewalskyi* Tschitschérine, 1 male (ZIN), N. E. Thibet, 1884, Przewalsky / Przewalskii m. typ. Tschitscherin det / Zoological Institute, Russian Academy of Sciences, St. Petersburg [yellow label]. 1 female (NMPC), N.E. Thibet, 1884, Przewalsky. / przewalskii m., typ., Tschitscherin det / Type [red label] / przewalskii, typ., Tsch., Det. Tschitscherin [pink label].

##### Non-type material.

**Figures 42–57.**
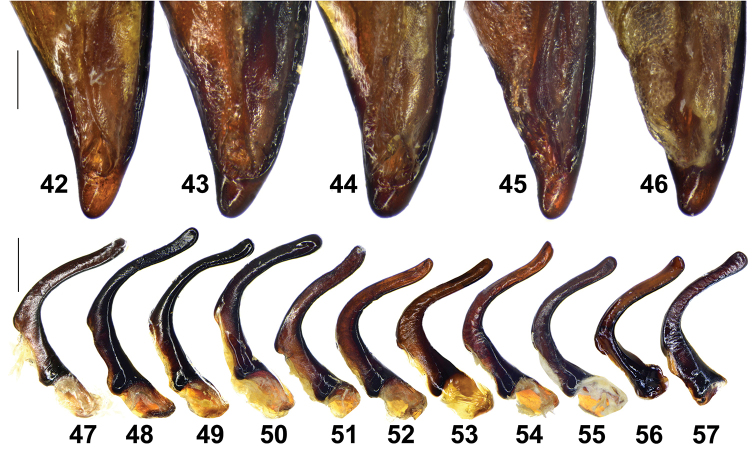
male genitalia of Pterostichus (Chinapterus) spp. **42–46** Apex of median lobe, dorsal left view **42***P. przewalskyi*, LT: Serxü (IZAS) **43***P. przewalskyi*, LT: Jigzhi (IZAS) **44***P. przewalskyi*, LT: Hongxing (IZAS) **45***P. lianhuaensis* sp. nov. Holotype (IZAS) **46***P. liupanensis* sp. nov. Holotype (IZAS) **47–57** right paramere, inner face **47–51***P. przewalskyi***52–54***P. lianhuaensis* sp. nov. **55–57***P. liupanensis* sp. nov. **47** Lectotype (ZIN) **48** LT: Jigzhi (IZAS) **49** LT: Jigzhi (IZAS) **50** LT: Hongxing (IZAS) **51** LT: Serxü (IZAS) **52** Holotype (IZAS) **53** Paratype, LT: Lianhuashan (IZAS) **54** Paratype, LT: Lianhuashan, Shahetan (IZAS) **55** Holotype (IZAS) **56** Paratype, LT: Liupanshan, Longtan (IZAS) **57** Paratype, LT: Liupanshan, Xixia (IZAS). Scale bars: 0.5 mm (**42–45**, **47–57**); 0.2 mm (**46**).

1 female (IZAS), China, N Sichuan prov., Hongyuan, ca. 4200 m, 21.7–3.8.1991, J. Kalab leg. 1 male (IZAS) [Figs 42, 51], Ch-NW Sichuan, ±3700 m, 32.59N, 98.06E, 3+15/7, SERXÜ env. 1995, alpine meadow, Jaroslav Turna leg. 1 female (IZAS), Qinghai, Baima county, Makehe, Meilanggou, 2013.VI.28, D 3500 m, Chen Jin leg. 2 males (IZAS) [Figs 25, 44, 50], Sichuan, Zoigê county, 8km W. of Hongxing, wetland, 3236 m, 34.1236N, 102.6545E, under rock, 2016.VII.25, Shi HL lgt. 3 males and 1 female (IZAS) [Figs 32, 33, 49], Qinghai, Jigzhi county, E. of Luanshitou pass; alpine meadow; 4091 m; 33.4008N, 101.2537E; 2017.VIII.2, pitfall trap, SHI HL, LU ZB, ZHU PZ & YAN WF leg. 1 male (IZAS) [Figs 43, 48], Qinghai, Jigzhi county, Luanshitou pass; alpine meadow; 4212 m; 33.3986N, 101.2432E; 2017.VIII.2, under stone, SHI HL, LU ZB, ZHU PZ & YAN WF leg. 8 females (IZAS) [Figs 59–61], Qinghai, Chidu county, 2 km S of Zhenqin; alpine wetland, 4329 m, 33.3908N, 97.2977E, 2017.VII.27, under stone, SHI HL, LU ZB, ZHU PZ & YAN WF leg.

##### Diagnosis.

Femora reddish brown. Pronotum cordate; lateral margins strongly sinuate before basal angles; basal angles right-angled, clearly pointed outwards. Elytral basal fovea depressed between inner and outer grooves; basal pore present; third interval often with two setigerous pores. Fifth tarsomere glabrous ventrally; right paramere strongly elongate and bent, apex rounded, not declined to dorsum.

##### Description.

BL 12.0–13.4 mm, BW 4.8–5.6 mm. Robust, black, femora reddish brown except black apex; elytra slightly shiny. ***Head*** large, frons smooth or very sparsely punctate; genae short, less than one-third length of eyes; eyes prominent. ***Pronotum*** cordate; widest slightly before middle, PW/PL = 1.33–1.41; lateral margins largely rounded before the middle, strongly sinuate before basal angles; one mid-lateral seta present at apical third; basal margin slightly wider than apical margin, PBW/PAW = 1.02–1.12; basal angles rectangular, clearly protruding outwards; basal foveae deep, inner and outer grooves faintly defined, inner groove straight, distant from basal margin, outer groove shorter than inner one, extending to basal margin; basal fovea depressed between inner and outer grooves, coarsely punctate in basal fovea; area between outer groove and lateral margin hardly convex; disc convex, smooth, slightly transversely rugose; apical angle rounded, not protruding. ***Elytra*** oblong, EL/EW = 1.42–1.45; basal ridge slightly oblique; shoulder rounded, basal ridge and lateral margin forming an obtuse angle, humeral tooth small and obtuse; apical plica indistinct; basal setigerous pores present; scutellar striae complete; intervals slightly convex; microsculpture similar in both sexes, finely isodiametric; third interval usually with two setigerous pores, at basal two-fifths and two-thirds respectively, occasionally with one or three pores on one elytron, all adjacent to second stria; ninth interval with umbilical series regularly arranged, slightly sparser in the middle; striae deep, indistinctly punctate. Fifth tarsomere glabrous ventrally; meso- and metatarsomere I and II with outer groove. Terminal ventrite shallowly depressed and finely rugose in males. ***Male genitalia.*** Ventral margin of median lobe nearly straight at middle, gradually bent downwards near apex; apical orifice opening left-dorsally; apical lamella small, rounded triangular, length sub-equal to basal width, apex rounded (Figs 30, 31); apical lamella slightly twisted, forming a continuously curved dorsal-left surface (Figs 42–44). Right paramere strongly elongate and curved, the obtuse angle between basal portion and apical portion 100–105°; apex slightly thick, well rounded, not or weakly bent to dorsum (Figs 47–51). Endophallus bent to the ventral side of aedeagus, major portion of endophallus located at ventral side of median lobe apex; gp opened to basal-dorsum; gpl large and coniform; gpp large and cap-like, on the ventral side of gp; two groups of lobes recognized: rl on ventral-right surface of endophallus, large, rounded or apex a little pointed; lbl on ventral-left surface of endophallus, divided into two clearly separated sub-lobes, the basal one large and nearly rounded, the apical one much smaller than the basal one; lal absent (Figs 32, 33). ***Female genitalia.*** Gonocoxite II of ovipositor slightly slender, length ca. 2.5 times maximum width; inner and outer margins near straight, each with one ensiform setae before middle, apex narrowly rounded, with two very short nematiform setae in a groove (Figs 60, 61). Spermatheca moderately long and tube-like, length ca. 15 times maximum width (Fig. 59).

##### Distribution.

**Map 2.**
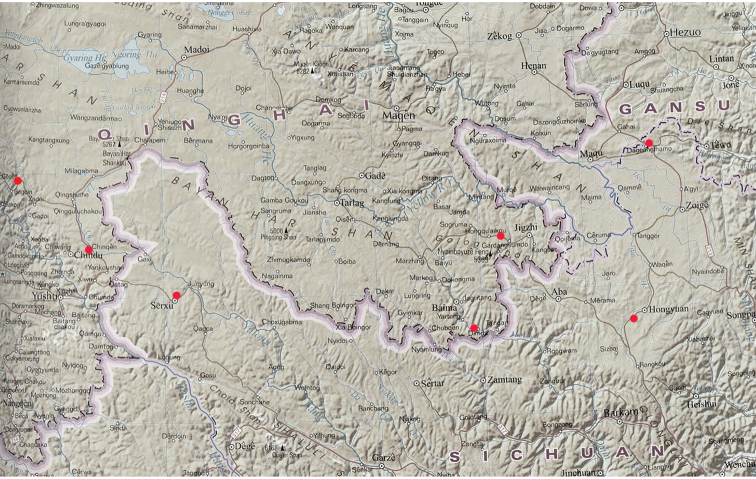
Distributions for *P. przewalskyi*.

Qinghai (southeast part), Sichuan (northwest part). Probably also in southwest Gansu (Map 2).

##### Habitat.

This hygrophilous species tends to live in wet habitats such as wetlands or the banks of seasonal streams on alpine meadows.

##### Remarks.

This species was described based on an unspecified number of specimens from “Amdo: près des rivières Tala-tchu et By-tchu” collected by Przewalsky in 1884. In the collection of ZIN, we examined two males which matched with the original literature. We designated the male with its genitalia dissected as the lectotype herein, for the taxonomic purpose of fixing the species name to a single specimen and preventing further confusion.

Sciaky (1996) assigned this species to the subgenus Sinoreophilus due to external similarities but this species has its right paramere strongly elongated and the elytral microsculpture is similar in both sexes, supporting a close relationship to *P. singularis*. So, we herein moved it from the subgenus Sinoreophilus to the subgenus Chinapterus.

#### Pterostichus (Chinapterus) lianhuaensis sp. nov.

Taxon classificationAnimaliaColeopteraCarabidae

3C9A21AD-7E1B-5957-880C-B5E946E930A1

http://zoobank.org/DE1CB5E1-EA91-4EDE-A244-70FC63F5DCFD

Habitus, Figs 26 , 27 ; male genitalia, Figs 34–37 , 45 , 52–54 ; female genitalia, Figs 61 , 62

##### Type series.

***Holotype***: male (IZAS) [Figs 26, 34, 35, 45, 52], China, Gansu, Kangle, Lianhuashan, Shahetan station 34.93917N, 103.73472E / 2850 m, 2008.VI.1, WANG J. leg.; pit fall; Institute of Zoology. ***Paratypes***: 1 male and 1 female (IZAS), 2012,VI,21 D 2960 m, China, Gansu, Kangle, Lianhuashan, 34.93577N, 103.75054E / Morii leg., Inst. of Zoology, CAS. 1 male and 2 females (IZAS) [Figs 27, 36, 37, 53, 62, 63], 2012,VI,22. D 2960 m, Gansu, Kangle, Lianhuashan, 34.91943N, 103.72860E / Liang Hongbin, Sota leg., Inst. of Zoology, CAS. 1 male and 1 female (IZAS) [Fig. 54], China, Gansu, Kangle, Lianhuashan, Shahetan station 34.93917N, 103.73472E / 2850 m, 2008.VII.6, WANG J. leg.; pitfall trap; Institute of Zoology.

##### Diagnosis.

Femora black; pronotum cordate, lateral margins strongly sinuate before basal angles, which clearly pointed outwards; basal foveae convex between inner and outer grooves; elytral basal pore present, third interval often with three setigerous pores; fifth tarsomere with one or two pairs of fine setae ventrally; right paramere strongly elongated and bent, apex slightly bent to dorsum, angulate at dorso-apical end.

##### Description.

BL 12.3–13.6 mm, BW 5.0–5.5 mm. Robust, black, femora completely black, elytra slightly shiny. Head large, frons smooth or very sparsely punctate; genae short, less than one-third length of eyes; eyes prominent. ***Pronotum*** cordate; widest slightly before middle, PW/PL = 1.31–1.40; lateral margins largely rounded before middle, strongly sinuate before basal angles; one mid-lateral seta present at apical one-third; basal margin slightly wider than apical margin, PBW/PAW = 1.02–1.13; basal angles rectangular, clearly protruding outwards; basal foveae narrow and deep, inner groove well present, apex reaching basal third of pronotum, outer groove obsolete; basal fovea convex between inner and outer grooves, not convex between outer groove and lateral margin, basal foveal area coarsely punctate; disc convex, smooth, finely transversely rugose on basal half; apical angles rounded, not protruding. ***Elytra*** oblong, EL/EW = 1.36–1.46; basal ridge slightly oblique; shoulder rounded, basal ridge and lateral margin forming an obtuse angle, humeral tooth small and obtuse; apical plica indistinct; basal setigerous pores present; scutellar striae complete; intervals slightly convex, microsculpture similar in both sexes, finely isodiametric; third interval usually with three setigerous pores, the basal one at basal seventh, adjacent to third stria, the apical two at basal two-fifths and two-thirds respectively, all adjacent to second stria, occasionally with four or five pores on one elytron; ninth interval with umbilical series regularly arranged, slightly sparser in middle; striae deep, indistinctly punctate. Fifth tarsomere with one or two pairs of setae ventrally; meso- and metatarsomere I and II with outer groove. Terminal ventrite shallowly depressed and finely rugose in males. ***Male genitalia.*** Ventral margin of median lobe near straight at middle, gradually bent downwards near apex; apical orifice opened left-dorsally; apical lamella small, rounded triangular, length subequal to basal width, apex rounded, weakly bent to left (Figs 34, 35); apical lamella slightly twisted, forming a continuously curved dorsal-left surface (Fig. 45). Right paramere strongly elongate and curved, the acute angle between basal portion and apical portion 85–90°; apex slightly thick, slightly angulate at dorsal-apical end, slightly bent to dorsum (Figs 52–54). Endophallus bent to the ventral side of aedeagus, major portion of endophallus located at ventral side of median lobe apex; gp opened to basal-dorsum; gpl large and coniform; gpp large and cap-like, on ventral side of gp; three groups of lobes recognized: rl on ventral-right surface of endophallus, large, near rounded, without projection; lbl on ventral-left surface of endophallus, large and fully round, not divided into sub-lobes; lal on left surface of endophallus, very small, rounded or divided into two small sub-lobes (Figs 36, 37). ***Female genitalia*** same as that of *P. przewalskyi* (Figs 62, 63).

##### Distribution.

**Map 3.**
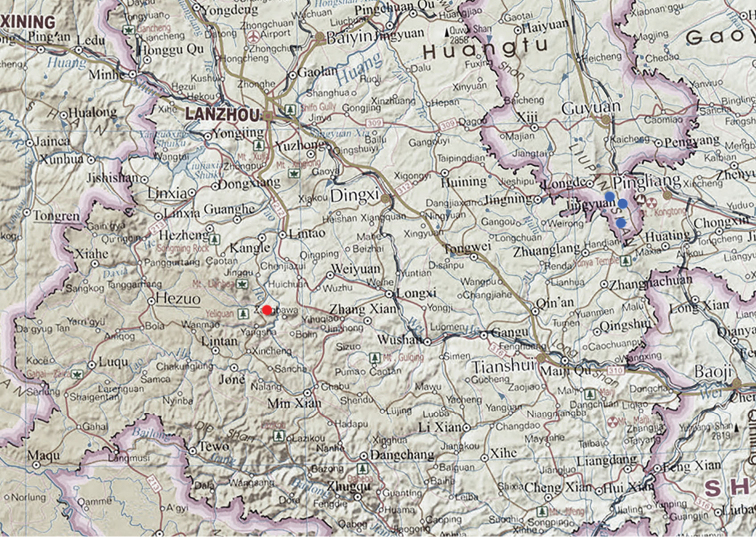
Distributions for *P. lianhuaensis* sp. nov. (red) and *P. liupanensis* sp. nov. (blue).

Only known from the type locality, Lianhua Shan mountain, Kangle County, south of Gansu province (Map 3).

##### Etymology.

The name of the new species refers to its type locality, Lianhua mountain.

##### Remarks.

This species is similar to *P. przewalskyi* in having a large body size, lateral margins of the pronotum strongly sinuate before basal angles, but differs from the latter in: (1) femora completely black; (2) basal foveae convex between inner and outer grooves; (3) elytra third interval usually with three pores, the basal one adjacent to the third stria; (4) fifth tarsomere with one or two setae ventrally. In *P. przewalskyi*, femora reddish brown; basal foveae of pronotum depressed between inner and outer grooves; elytral third interval usually with two pores, if with three the basal one adjacent to the second stria; fifth tarsomere glabrous ventrally. The male genitalia of these two species are very similar, with small differences in: (1) apical lamella of aedeagus weakly bent to left in *P. lianhuaensis* sp. nov. (Fig. 34), almost straight in *P. przewalskyi* (Fig. 30); (2) apex of right paramere slightly angulate at dorsal-apical end in *P. lianhuaensis* sp. nov., completely rounded in *P. przewalskyi*; (3) right paramere more bent in *P. lianhuaensis* sp. nov., forming an acute angle 85–90°, versus 100–105° in *P. przewalskyi*; (4) endophallus with lbl not divided and lal present in *P. lianhuaensis* sp. nov., lbl divided into two sub-lobes and lal absent in *P. przewalskyi*.

We noticed that this new species only has very minute differences from *P. przewalskyi* in the male genitalia. However, considering the stable gaps between their features (especially the external characters) and distributions, we decide to establish a new species but not a new subspecies. A similar situation is also present in the next new species, *P. liupanensis*. Several external differences among these three species are generally of interspecific level in other *Pterostichus* groups, such as the shape of the pronotum base and basal fovea, chaetotaxy on elytral third interval, and setae on the fifth tarsomere.

#### Pterostichus (Chinapterus) liupanensis sp. nov.

Taxon classificationAnimaliaColeopteraCarabidae

73854F7C-6587-561E-BCC8-EE2DB2AA9397

http://zoobank.org/56C5BFFA-CAD8-41A5-BF61-A39BFEB7B862

Habitus, Figs 28 , 29 ; male genitalia, Figs 38–41 , 46 , 55–57

##### Type series.

***Holotype***: male (IZAS) [Figs 28, 38, 39, 46, 55], China, Ningxia A.R., Liupanshan, Xixia Forestry Station, 35.51620N, 106.25086E / 2200 m, 2008.6.25–27, Zhao Zongyi Pitfall trap, Institute of Zoology. ***Paratypes***, 1 male (IZAS) [Fig. 56], China, Ningxia A.R., Liupanshan, Longtan Forestry Station, 35.38982N, 106.34508E / 1936 m, 2008.6.23–25, Lou Qiaozhe, pitfall trap, Institute of Zoology. 1 male (IZAS) [Figs 29, 40, 41, 57], China, Ningxia A.R., Liupanshan, Xixia Forestry Station, 35.49673N, 106.31310E / 1994 m, 2008.6.25N, Lou Qiaozhe collector, Institute of Zoology.

##### Diagnosis.

Femora black; pronotum near quadrate, lateral margins near straight before basal angles, which weakly pointed outward and with a small denticle; basal foveae convex between inner and outer grooves; elytral basal pore present, third interval usually with three setigerous pores; fifth tarsomere glabrous ventrally; right paramere strongly elongated and bent, apex rounded, not bent to dorsum.

##### Description.

BL 12.4–13.2 mm, BW 4.8–5.1 mm. Robust, black, femora black, elytra slightly shiny. Head large, frons smooth or very sparsely punctate; genae short, less than one-third length of eyes; eyes prominent. ***Pronotum*** near quadrate; widest slightly before middle, PW/PL = 1.33–1.37; lateral margins largely rounded before middle, nearly straight before basal angles; one mid-lateral seta present at apical one-third; basal margin slightly wider than apical margin, PBW/PAW = 1.12–1.16; basal angles rectangular, weakly protruding outwards, with a small denticle; basal foveae narrow and deep, inner groove obviously present, apex reaching basal third of pronotum, outer groove obsolete; basal fovea convex between inner and outer grooves, not convex between outer groove and lateral margin, basal foveal area coarsely punctate; disc convex, smooth, finely transversely rugose at basal half; apical angle rounded, not protruding. ***Elytra*** oblong, EL/EW = 1.46–1.52; basal ridge slightly oblique; shoulder rounded, basal ridge and lateral margin forming an obtuse angle, humeral tooth small and obtuse; apical plica indistinct; basal setigerous pores present; scutellar striae complete; intervals slightly convex, microsculpture finely isodiametric in males; third interval with three setigerous pores, the basal one at basal seventh, adjacent to third stria, the apical two at middle and apical fourth respectively, all adjacent to second stria; ninth interval with umbilical series regularly arranged, slightly sparser in middle; striae deep, indistinctly punctate. Fifth tarsomere glabrous ventrally; meso- and meta-tarsomeres I and II with outer groove. Terminal ventrite shallowly depressed and rugose in males. ***Male genitalia.*** Ventral margin of median lobe near straight at middle, obviously bent downwards near apex; apical orifice opened left-dorsally; apical lamella small, rounded triangular, length sub-equal to basal width, apex rounded (Figs 38, 39); apical lamella not twisted with its dorsal surface perpendicular to left surface (Fig. 46). Right paramere strongly elongate and curved, the acute angle between basal portion and apical portion near 90°; apex slightly thick, well rounded, not or weakly bent to dorsum (Figs 55–57). Endophallus bent to the ventral side of aedeagus, major portion of endophallus located at ventral side of median lobe apex; gp opened to basal-dorsum; gpp large and cap-like, on ventral side of gp; three groups of lobes recognized: rl on ventral-right surface of endophallus, large and rounded, with a papillary projection to the apex of endophallus; lbl on ventral-left surface of endophallus, divided into two clearly separated sub-lobes, the apical one very small, same size as lal, the basal one large, same size as rl; lal on left surface of endophallus, very small and rounded (Figs 40, 41).

##### Distribution.

Only known from the Liupan Shan mountain range, Jingyuan County, Ningxia Huizu Autonomous Region (Map 3).

##### Etymology.

The name of the new species refers to its type locality, Liupan Shan mountain.

**Map 4.**
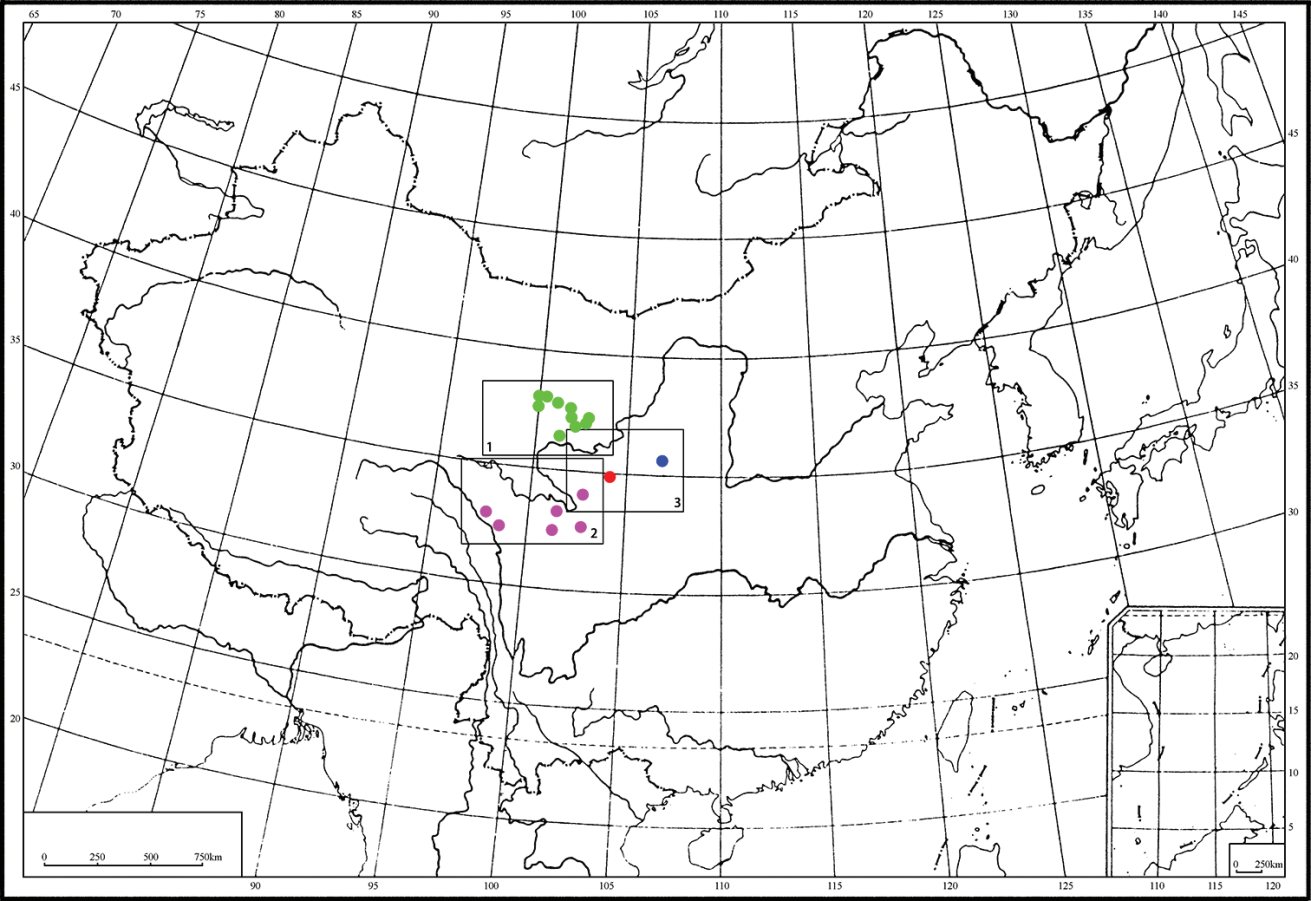
Distributions for Pterostichus (Chinapterus) spp. *P. singularis* (green), *P. przewalskyi* (violet), *P. lianhuaensis* sp. nov. (red), *P. liupanensis* sp. nov. (blue); black boxes mark area shown in maps 1–3.

##### Remarks.

The new species is similar to *P. lianhuaensis* sp. nov. in having a large body size, black femora, narrow pronotum basal fovea, elytra third interval usually with three pores, but differs from the latter by having: (1) pronotum lateral margins nearly straight before basal angles; and (2) fifth tarsomere glabrous ventrally. In *P. lianhuaensis* sp. nov., pronotum lateral margins strongly sinuate before basal angles; fifth tarsomere with one or two pairs of setae ventrally. The male genitalia of the new species is very similar to that of *P. przewalskyi*, with small differences in: (1) ventral margin of aedeagus more distinctly bent downwards near apex in lateral view in *P. liupanensis* sp. nov. (Fig. 39), evenly and gradually bent downwards in lateral view in *P. przewalskyi* (Fig. 31); (2) apical lamella with dorsal surface perpendicular to left surface in *P. liupanensis* sp. nov., slightly twisted, forming a continuously curved dorsal-left surface in *P. przewalskyi*; (3) right paramere more bent forming an acute angle near 90°, versus 100–105° in *P. przewalskyi*; (4) endophallus with rl projected to the apex of endophallus and lal present, in *P. przewalskyi*rl not or slightly projected to the base of endophallus and lal absent.

**Figures 58–65.**
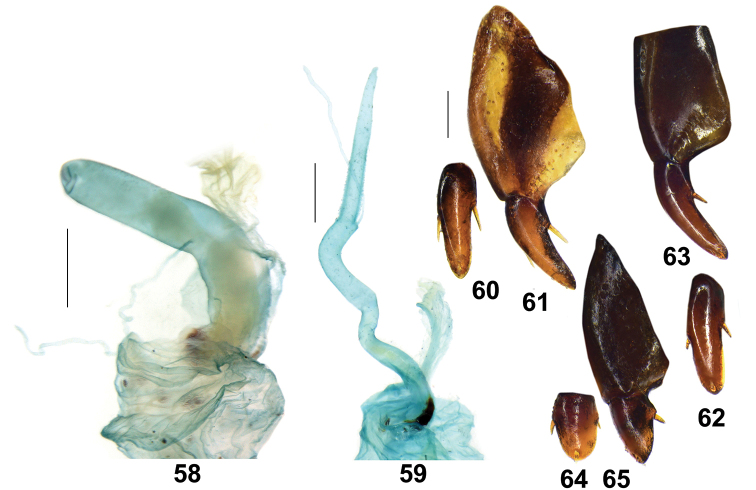
female genitalia of Pterostichus (Chinapterus) spp. **58, 59** Female reproductive system **58***P. singularis*, LT: Pass Xining-Guide (IZAS) **59***P. przewalskyi*, LT: Zhenqin (IZAS). Scale bar: 0.5 mm. **60–65** Female ovipositor **60, 61***P. przewalskyi*, LT: Zhenqin (IZAS) **62, 63***P. lianhuaensis* sp. nov., Paratype LT: Lianhuashan (IZAS) **64–65***P. singularis*, LT: Pass Xining-Guide (IZAS) **60, 62, 64** Gonocoxite II, inner view, **61, 63, 65** Gonocoxite I, II, ventral view. Scale basr: 0.2 mm.

## Supplementary Material

XML Treatment for Subgenus Chinapterus

XML Treatment for Pterostichus (Chinapterus) singularis

XML Treatment for Pterostichus (Chinapterus) przewalskyi

XML Treatment for Pterostichus (Chinapterus) lianhuaensis

XML Treatment for Pterostichus (Chinapterus) liupanensis
